# Reference range of neutrophil-to-lymphocyte ratio in healthy individuals and its predictive value for post-trauma nosocomial infections

**DOI:** 10.3389/fcimb.2025.1529532

**Published:** 2025-04-22

**Authors:** Qiang Wang, Yu Jiang, Feifei Jin, Lebin Gan, Rui Li, Diya Sun, Mengwei Zhang, Zishuo Pei, Jingyao Zhang, Jingjing Ye, Tianbing Wang

**Affiliations:** ^1^ Trauma Treatment Center, Peking University People’s Hospital, Key Laboratory of Trauma Treatment and Neural Regeneration (Peking University) Ministry of Education, National Center for Trauma Medicine, Beijing, China; ^2^ Department of Clinical Laboratory, Chengdu Integrated TCM & Western Medicine Hospital, Chengdu, China

**Keywords:** NLR, trauma, infection, risk factors, reference range

## Abstract

**Background:**

This study aimed to establish the reference range of the neutrophil-to-lymphocyte absolute ratio (NLR) in healthy individuals, explore the association between NLR and post-trauma nosocomial infections, and evaluate the effectiveness of NLR in predicting clinical outcomes of post-trauma infections.

**Methods:**

A retrospective analysis was conducted based on medical records of acute trauma patients from the China National Center for Trauma Medical and health examination data from Chengdu First People’s Hospital, Sichuan Province. The reference range of NLR was established, and multivariate logistic regression analysis was performed to identify risk factors for infection, with subgroup analysis conducted by age. The predictive value of NLR for post-trauma infections was assessed using the area under the receiver operating characteristic curve (AUC).

**Results:**

A total of 175,019 individuals were included, comprising 165,504 healthy individuals (753 minors [0.45%, <18 years] and 164,751 adults [99.55%]) and 9,515 acute trauma patients (8,602 in the control group [90.40%] and 913 with post-trauma infections [9.60%]). The 2.5th-97.5th percentile range for NLR was 0.69-3.48 in minors and 0.86-3.83 in adults. NLR was identified as an independent risk factor for post-trauma infections, with an odds ratio (OR) of 1.43 (95% CI: 1.18-1.73). Additionally, NLR showed a positive correlation with common inflammatory markers, CRP (r = 0.37 [0.32-0.42], weak correlation) and PCT (r = 0.52 [0.45-0.58], moderate correlation). Receiver operating characteristic (ROC) analysis demonstrated that NLR had an AUC of 0.71 (95% CI: 0.69-0.73, *P* < 0.0001) for predicting infections, with a diagnostic cutoff value of 4, sensitivity of 60.28% (95% CI: 59.24-61.31), and specificity of 72.85% (95% CI: 69.71-75.77). No significant differences in NLR values were observed between groups with pulmonary infections, urinary tract infections, combined pulmonary and urinary infections, and soft tissue infections (*P* > 0.05). However, NLR values were significantly higher in patients with fungal infections compared to those without (*P* = 0.03).

**Conclusion:**

The current 2.5th-97.5th percentile range of NLR is 0.69-3.48 in minors and 0.86-3.83 in adults. With increasing age, the reference range widens, and females tend to have a slightly broader range than males. NLR is an independent risk factor for post-trauma infections, showing higher predictive value in patients over 60 years of age. Although NLR cannot differentiate infection sites or pathogen types, elevated NLR values may provide a reference for identifying fungal infections.

## Introduction

Trauma is a leading cause of morbidity and mortality among individuals under the age of 45 Worldwide (Lozano et al., 2012). The occurrence of nosocomial infections following trauma further exacerbates the burden of trauma on patient health and healthcare systems (Lozano et al., 2012; Weir et al., 2010; Jiang et al., 2017; Fares et al., 2013; Glance et al., 2011). Studies indicate that approximately 4% of hospitalized patients experience at least one nosocomial infection, with the most common types including pneumonia (21.8%), surgical site infections (21.8%), gastrointestinal infections (17.1%), urinary tract infections (12.9%), and primary bloodstream infections (9.9%). These infections significantly prolong hospital stays, increase medical costs, and elevate mortality rate (Glance et al., 2011; Macaluso, 2023; Magill et al., 2021; Danna, 2018; Thaden et al., 2017; Vincent et al., 2020). In trauma patients, post-traumatic pneumonia increases mortality by 5- to 20-fold, with rates reaching as high as 30% in some regions (Morrow et al., 2022).

Trauma-induced tissue and cellular damage triggers a complex immune response, resulting in the release of damage-associated molecular patterns (DAMPs) and a rapid surge of inflammatory mediators. To prevent excessive inflammation, the body activates compensatory mechanisms to balance pro-inflammatory and anti-inflammatory responses. However, disruption of this balance can lead to prolonged immunosuppression, increasing the risk of secondary infections. Therefore, monitoring and evaluating early immune status is critical for predicting infection risk (Tuano et al., 2021). The neutrophil-to-lymphocyte ratio (NLR) is an important marker of inflammatory and immune Status (Korkmaz et al., 2015). Numerous studies have demonstrated that NLR is a rapid and effective immunological biomarker with significant utility in various inflammatory and immune conditions (Howard et al., 2019; Cupp et al., 2020; Farkas, 2020; Marik and Stephenson, 2020; Ham et al., 2020). NLR reflects the dynamic balance between innate and adaptive immune responses, making it a valuable indicator of the interaction between innate immunity and acquired immunity (Morrow et al., 2022). NLR has been widely applied in the diagnosis and assessment of multiple inflammation-related diseases (Korkmaz et al., 2015).

However, the reference range of NLR in healthy populations and its role in predicting nosocomial infections in trauma patients remain underexplored. This study aims to establish the reference range of NLR in healthy individuals and investigate its potential value in predicting nosocomial infections following trauma. By supplementing existing knowledge on early risk factors for post-traumatic infections, this study seeks to provide new insights for the early detection, prevention, and intervention of trauma-related infections, ultimately reducing nosocomial infection rates and alleviating the burden of trauma on patients and healthcare systems.

## Methods

### Data sources and study design

This retrospective study included data from two sources: the electronic medical records database of the China National Center for Trauma Medical (Peking University People’s Hospital) and the health examination database of Chengdu First People’s Hospital in Sichuan Province, China. Data from the China National Center for Trauma Medical were collected from January 1, 2012, to April 1, 2023, while data from Chengdu First People’s Hospital were collected from January 1, 2023, to October 1, 2024. The dataset included demographic information and laboratory results of the health examination population, as well as inpatient records and initial clinical laboratory results of trauma patients. The study first used data from the health examination population to establish the reference range for NLR. Subsequently, nosocomial infection in trauma patients from the National Trauma Medical Center was used as the outcome variable. Multivariate logistic regression analysis was employed to investigate the association between various independent variables and the outcome variable.

Inclusion criteria for trauma patients were: (1) age >18 years; (2) acute trauma; and (3) hospitalization duration >24 hours. Nosocomial infection was defined as infections acquired during hospitalization that were not present or incubating at the time of admission, including infections that became evident after discharge but were acquired during the hospital stay (Garner et al., 1988). This study was approved by the Ethics Committees of Peking University People’s Hospital and Chengdu First People’s Hospital (Approval Nos. 2024PHB136-001 and 2024-YNYJ-030). The study adhered to the Strengthening the Reporting of Observational Studies in Epidemiology (STROBE) guidelines, with the full checklist provided in **Supplementary Table 1**.

### Variable types and missing data handling

Based on the clinical experience of two trauma center staff members (Pei Zishuo and Zhang Mengwei) and existing literature on post-trauma infections, we identified and selected clinical variables for inclusion in the study. These variables encompass basic clinical information, underlying diseases, laboratory test results, and other factors (e.g., use of mechanical ventilation and urinary catheterization). All laboratory test results were defined as the first recorded values after patient admission.

Missing Data Handling Principles: For the study variables, any variable with a missing rate exceeding 10% was excluded to minimize bias. For binary variables, the majority category was used to impute missing data to reduce potential bias. For continuous numerical variables, missing values were imputed using the mean of the respective variable.

### Statistical methods

Categorical variables were expressed as frequencies and proportions, while continuous variables were presented as mean ± standard deviation or median with interquartile range (IQR). For data not following a normal distribution, non-parametric tests were used for analysis. Multiple group comparisons were conducted using rank-based one-way analysis of variance (ANOVA). The reference intervals for the NLR were determined based on the 2.5th-97.5th percentile range of the dataset. Non-parametric rank correlation analysis was employed for linear correlation analyses. Identification of Risk Factors: Risk factors were identified using the least absolute shrinkage and selection operator (LASSO) method, followed by univariate analysis of independent and outcome variables. Variables with significant results in univariate analysis were further analyzed using multivariate logistic regression to identify independent risk factors. The procedure included: Using LASSO regression to screen predictors of post-trauma infections and identify potential influencing factors. Conducting multivariate logistic regression analysis to evaluate the impact of these factors on the risk of post-trauma infection. Calculating odds ratios (OR) and corresponding p-values for each variable. Variables with p-values <0.05 were considered independent influencing factors, with OR >1 indicating independent risk factors and OR <1 indicating independent protective factors. Subgroup analyses for independent influencing factors were performed using the same screening methods. Evaluation of Predictive Ability: The predictive ability of independent risk factors for post-trauma infections was evaluated by constructing receiver operating characteristic (ROC) curves and calculating the area under the curve (AUC). An AUC >0.7 indicated good predictive ability. The cutoff value, sensitivity, and specificity were also determined and compared with commonly used inflammatory markers in trauma. Subgroup comparisons were visualized using violin plots to display medians, IQRs, and intergroup statistical differences. The statistical analysis was performed using Prism 8.0 and R version 4.4.1, while Adobe Illustrator 2021 was used for graphical representation. A *P <*0.05 was considered statistically significant.

## Results

### Basic characteristics of the study population

**Figure 1:**
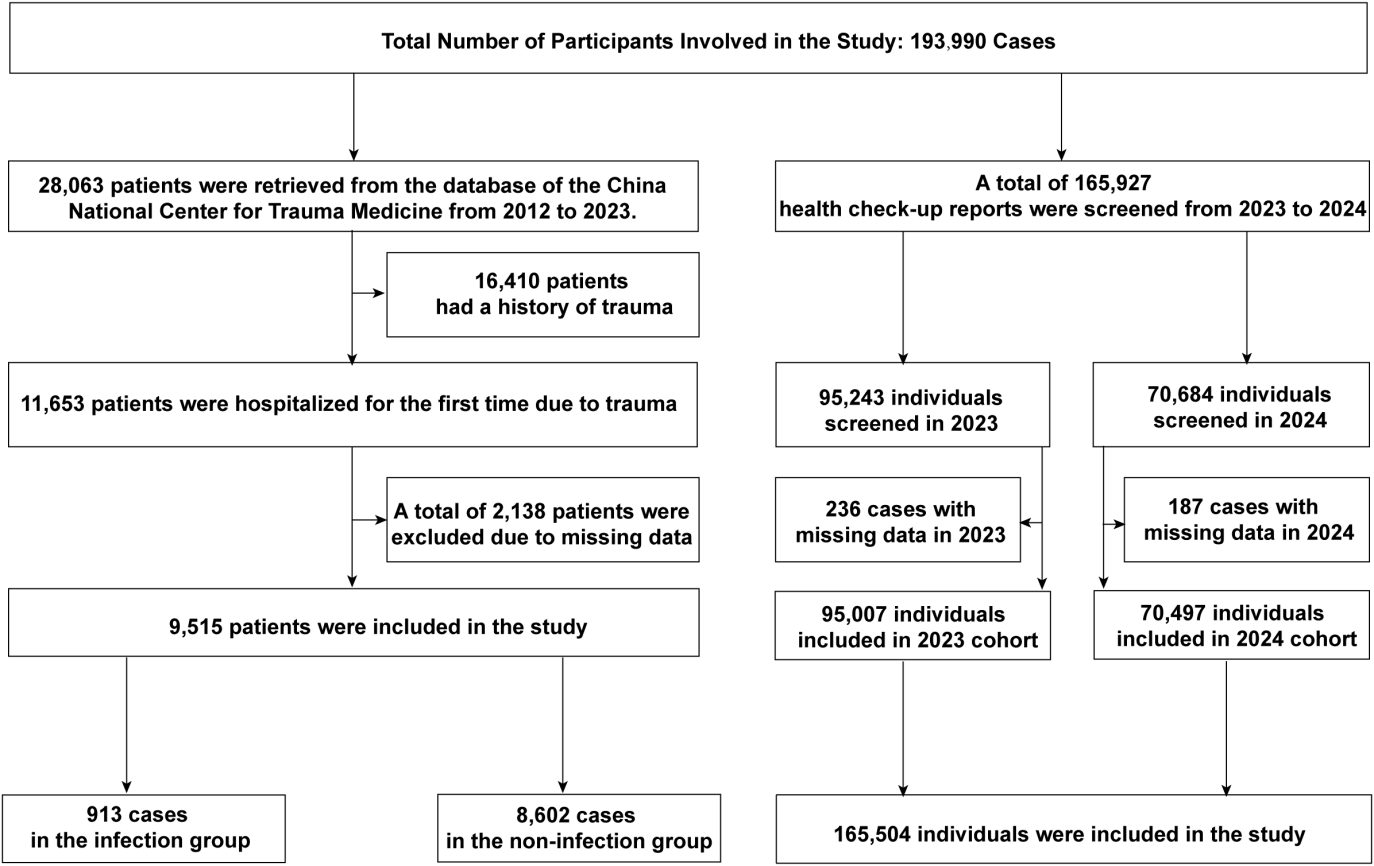
Selection of study patients.

A total of 193,990 individuals were included in this study. Data from the electronic medical records of the National Trauma Medical Center comprised 28,063 hospitalized patients, of which 9,515 acute trauma patients were included after screening. Among them, 8,602 patients (90.40%) were in the control group, and 913 patients (9.60%) in the experimental group developed post-trauma infections. The median age of the study population was 59 years (IQR: 40-74). Post-trauma infections accounted for 3.47% of all hospitalized patients during the study period and 9.60% of acute trauma admissions. The types of infections included: Pulmonary infections: 668 cases (73.17%), Urinary tract infections: 105 cases (11.50%), Combined pulmonary and urinary infections: 47 cases (5.15%), Soft tissue (wound) infections: 46 cases (5.04%), Other infections: 47 cases (5.15%). Among infected patients, 31 (3.40%) died during hospitalization, compared to 37 deaths (0.43%) in the non-infected group. The primary pathogens were bacteria, with fungal coinfections in 8 cases (0.88% of the infected group). Eighteen patients (1.97%) developed septic shock. Blood cultures were performed in 80 patients, all of which returned negative results. The control group had an average mechanical ventilation duration of 105.00 hours, an average ICU stay of 6.46 days, an average hospital stay of 9.02 days, and an average hospitalization cost of 56,326 CNY. In contrast, the experimental group had significantly higher values: an average ventilation duration of 235.30 hours, an average ICU stay of 12.34 days, an average hospital stay of 15.63 days, and an average hospitalization cost of 90,209 CNY (**Figure 1**; **Table 1**).

**Table 1:** Clinical characteristics of the traumatic patients included in the study.

Variables		No. (%) of patients			P value
		Total	Non infectious group	Infectious group	
		(n = 9515)	(n = 8602)	(n = 913)	
Sex	n (%)				< 0.001
Female		4950 (48.24)	4200 (48.83)	548 (60.02)	
Male		4565 (51.76)	4402 (51.17)	365 (39.98)	
Hypertension	n (%)				< 0.001
No		7123 (74.86)	6614 (76.89)	509 (55.75)	
Yes		2392 (25.14)	1988 (23.14)	404 (44.25)	
Diabetes	n (%)				< 0.001
No		8324 (87.48)	7623 (88.62)	701 (76.78)	
Yes		1191 (12.52)	979 (11.38)	212 (23.22)	
Cerebral_infarction	n (%)				< 0.001
No		9068 (95.30)	8271 (96.15)	797 (87.29)	
Yes		447 (4.70)	331 (3.85)	116 (12.21)	
CHD	n (%)				0.003
No		9242 (97.13)	8370 (97.30)	872 (95.51)	
Yes		273 (2.87)	232 (2.70)	41 (4.49)	
Smoking	n (%)				0.71
No		8653 (90.94)	7819 (90.90)	834 (91.35)	
Yes		862 (9.06)	783 (9.10)	79 (8.65)	
Drinking	n (%)				0.673
No		8723 (91.68)	7882 (91.63)	841 (92.11)	
Yes		792 (8.32)	720 (8.37)	72 (7.89)	
Catheterization	n (%)				< 0.001
No		5627 (59.14)	5361 (62.32)	266 (29.13)	
Yes		3888 (40.86)	3241 (37.68)	647 (70.87)	
Ventilator	n (%)				< 0.001
No		9124 (95.89)	8295 (96.43)	829 (90.80)	
Yes		391 (4.11)	307 (3.57)	84 (9.20)	
shock	n (%)	126 (1.32)	37 (0.43)	89 (9.74)	< 0.001
MODs	n (%)	21 (0.22)	8 (0.09)	13 (1.42)	< 0.001
Hospital mortality	n (%)	68 (0.71)	37 (0.43)	31 (3.40)	< 0.001
Duration of ventilator (h)	Median (Q1,Q3)	132.9 (7,179)	105.0 (5,142.8)	235.3 (35,331)	< 0.001
Hospitalization expenses	Median (Q1,Q3)	59575 (23776,72684)	56326 (21286,69434)	90209 (51634,10358)	< 0.001
Hospitalization days	Median (Q1,Q3)	9.65 (4.82,11.89)	9.02 (4.68,11.38)	15.63 (7.99,17.78)	< 0.001
ICU length of stay	Median (Q1,Q3)	7.88 (0.99,10.64)	6.46 (0.98,8.62)	12.34 (0.99,15.85)	< 0.001
Injury types					
High falling injury	n (%)	178 (1.87)	161 (1.87)	17 (1.86)	
Traffic accident injury	n (%)	411 (4.32)	370 (4.30)	41 (4.49)	
Fall injury	n (%)	2325 (24.44)	2000 (23.25)	325 (35.60)	
Others	n (%)	6601 (69.37)	6071 (70.58)	530 (58.05)	
Age	Median (Q1,Q3)	59 (40, 74)	57 (39, 71)	77 (64, 85)	< 0.001
WBC	Median (Q1,Q3)	7.4 (5.92, 9.16)	7.3 (5.9, 9.07)	7.94 (6.59, 10.2)	< 0.001
RBC	Median (Q1,Q3)	4.17 (3.73, 4.6)	4.2 (3.77, 4.62)	3.9 (3.39, 4.22)	< 0.001
HB	Median (Q1,Q3)	129 (116, 142)	130 (117, 143)	122 (106, 130)	< 0.001
PLT	Median (Q1,Q3)	203 (166, 243)	204 (167, 245)	195 (151, 231)	< 0.001
LY	Median (Q1,Q3)	1.41 (1.04, 1.83)	1.48 (1.1, 1.9)	1.11 (0.8, 1.55)	< 0.001
MO	Median (Q1,Q3)	0.51 (0.4, 0.7)	0.5 (0.4, 0.7)	0.57 (0.45, 0.74)	< 0.001
NE	Median (Q1,Q3)	5.1 (3.8, 6.8)	5 (3.7, 6.7)	5.8 (4.8, 8.12)	< 0.001
NLR	Median (Q1,Q3)	3.55 (2.27, 5.64)	3.36 (2.19, 5.33)	5.28 (3.66, 8.45)	< 0.001

Data from Chengdu First People’s Hospital included 165,927 individuals undergoing health examinations, of which 165,504 were included after screening. Among these, 753 (0.45%) were minors (<18 years), and 164,751 (99.55%) were adults. In 2023, 95,007 individuals were included, with a male-to-female ratio of approximately 1.3:1. The median age was 35 years (IQR: 28-47), with a range of 3 to 98 years. The median neutrophil count (NE) was 3.36 (IQR: 2.73-4.13), lymphocyte count (LY) was 1.92 (IQR: 1.59-2.31), and NLR was 1.74 (IQR: 1.37-2.22). In 2024, 70,497 individuals were included, with a male-to-female ratio of approximately 1.2:1. The median age was 35 years (IQR: 28-46), with a range of 4 to 94 years. The median NE was 3.40 (IQR: 2.76-4.18), LY was 1.96 (IQR: 1.62-2.36), and NLR was 1.73 (IQR: 1.36-2.21) (**Figure 1**; **Table 2**).

**Table 2:** Baseline characteristics of the health check-up population.

Years	Variables		Age Stratification (years)				
			ALL	< 18	18-40	41-60	≥ 61
2023	Sex	n (%)					
	Male		53348 (56.15)	213 (50.59)	35145 (56.67)	14618 (56.10)	3372 (51.70)
	Female		41659 (43.85)	208 (49.41)	26870 (43.33)	11442 (43.90)	3139 (48.30)
	Age (years)	Median (Q1,Q3)	35 (28,47)	16 (14,17)	30 (26,34)	50 (45,55)	69 (65,75)
	NE (×109/L)	Median (Q1,Q3)	3.36 (2.73,4.13)	3.20 (2.66,4.05)	3.38 (2.75,4.15)	3.31 (2.68,4.09)	3.32 (2.71,4.06)
	LY (×109/L)	Median (Q1,Q3)	1.92 (1.59,2.31)	2.24 (1.88,2.77)	1.97 (1.64,2.36)	1.82 (1.49,2.19)	1.81 (1.45,2.23)
	NLR	Median (Q1,Q3)	1.74 (1.37,2.22)	1.45 (1.08,1.86)	1.71 (1.35,2.16)	1.82 (1.43,2.33)	1.83 (1.40,2.41)
2024	Sex	n (%)					
	Male		38588 (54.74)	196 (59.04)	25288 (54.22)	10486 (57.29)	2618 (50.14)
	Female		31909 (45.26)	136 (40.96)	21353 (45.78)	7817 (42.71)	2603 (49.86)
	Age (years)	Median (Q1,Q3)	35 (28,46)	16 (14,17)	30 (26,35)	50 (44,54)	68 (63,73)
	NE (×109/L)	Median (Q1,Q3)	3.40 (2.76,4.18)	3.42 (2.81,4.28)	3.44 (2.79,4.22)	3.33 (2.70,4.11)	3.30 (2.69,4.05)
	LY (×109/L)	Median (Q1,Q3)	1.96 (1.62,2.36)	2.40 (1.97,2.84)	2.00 (1.66,2.40)	1.86 (1.53,2.25)	1.86 (1.51,2.28)
	NLR	Median (Q1,Q3)	1.73 (1.36,2.21)	1.45 (1.11,1.88)	1.71 (1.35,2.17)	1.79 (1.40,2.28)	1.78 (1.36,2.33)

### Variables and missing data handling for the trauma population

Post-trauma infection status was used as the dependent variable, while the neutrophil-to-lymphocyte ratio (NLR), calculated from absolute neutrophil and lymphocyte counts, was included as one of the independent variables. Additional clinical variables were also incorporated, resulting in a total of 18 independent variables for the correlation analysis with post-trauma infections. These variables included: age (≥18 years), sex (male or female), smoking and alcohol consumption history, comorbidities (e.g., hypertension, diabetes, coronary artery disease, cerebral infarction), and laboratory test results (white blood cell count, red blood cell count, platelet count, hemoglobin level, absolute lymphocyte count, absolute neutrophil count, and absolute monocyte count). All laboratory test results were based on the first measurements obtained after hospital admission. Among the 18 variables, 9 were continuous numerical variables, and 9 were binary categorical variables. Of these, 9 variables had no missing data, while 7 variables had less than 2% missing data. Smoking and alcohol consumption history had the highest proportion of missing values, each accounting for approximately 6% of the total for their respective variables. According to our data handling principles, missing values in continuous variables were imputed using the mean of the respective variable, while missing values in binary variables were imputed using the most frequent category within the variable (**Supplementary Figure 1**).

### Preliminary determination of NLR reference range in the population

Data from health examination participants at Chengdu First People’s Hospital in 2023 and 2024 were used to establish the 2.5th–97.5th percentile range for NLR. For minors, the mean NLR was 1.59 ± 0.83, with a 2.5th–97.5th percentile range of 0.69-3.48. The median and interquartile range (IQR) were 1.45 (1.10-1.88), with a minimum value of 0.51 and a maximum value of 13.00. For adults, the mean NLR was 1.89 ± 0.84, with a 2.5th–97.5th percentile range of 0.86-3.83. The median and IQR were 1.74 (1.37-2.21), with a minimum value of 0.04 and a maximum value of 33.60. A statistically significant difference was observed between minors and adults (*P* < 0.0001), with the mean NLR of adults being 0.29 higher than that of minors. NLR in Adults by Age Group: 18-40 years: Mean NLR was 1.85 ± 0.81, with a 2.5th–97.5th percentile range of 0.85-3.70. The median and IQR were 1.71 (1.35-2.16), with a minimum value of 0.17 and a maximum value of 33.60. 41-60 years: Mean NLR was 1.96 ± 0.87, with a 2.5th–97.5th percentile range of 0.89-3.97. The median and IQR were 1.81 (1.42-2.31) with a minimum value of 0.30 and a maximum value of 28.86. ≥61 years: Mean NLR was 1.99 ± 0.99, with a 2.5th–97.5th percentile range of 0.83-4.31. The median and IQR were 1.80 (1.38-2.36), with a minimum value of 0.04 and a maximum value of 31.71. Significant differences were found when comparing the 18-40 age group to the 41-60 and ≥61 groups (*P* < 0.0001), with the mean NLR in the 18-40 age group being 0.11 and 0.14 lower than the other two groups, respectively. No statistically significant difference was found between the 41-60 and ≥61 age groups (*P* = 0.42). NLR in Adults by Gender: Males: Mean NLR was 1.87 ± 0.82, with a 2.5th–97.5th percentile range of 0.86-3.76. The median and IQR were 1.72 (1.36-2.19), with a minimum value of 0.014 and a maximum value of 31.71. Females: Mean NLR was 1.91 ± 0.87, with a 2.5th–97.5th percentile range of 0.85-3.90. The median and IQR were 1.76 (1.38-2.25), with a minimum value of 0.17 and a maximum value of 33.60. A statistically significant difference was observed between males and females (*P* < 0.0001), with females having a mean NLR 0.04 higher than males.

### Correlation analysis between age and NLR

**Figure 2:**
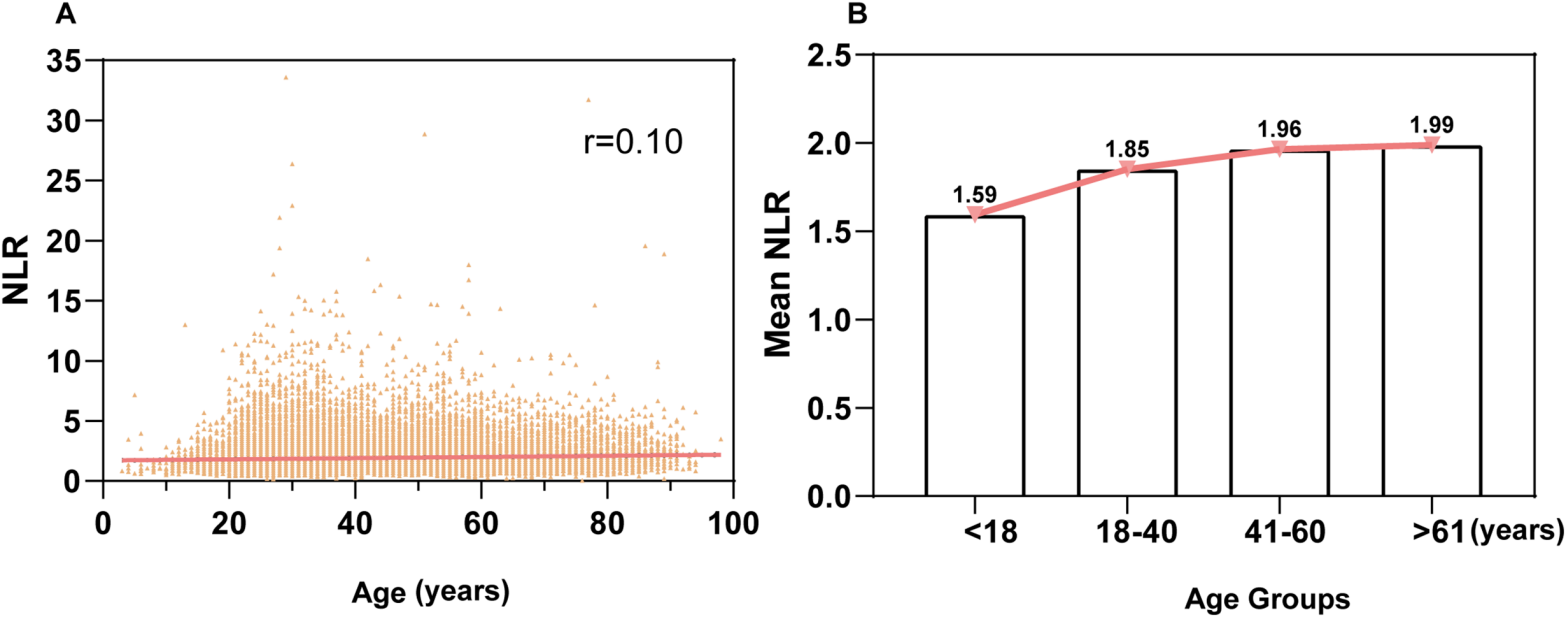
Correlation analysis of the relationship between age and NLR **(A)**: Linear correlation analysis between age and NLR in the entire population. The correlation coefficient (r) is 0.10, indicating no linear correlation. **(B)**: Relationship between age groups and NLR. The population was stratified into four age groups: <18 years, 18-40 years, 41-60 years, and ≥61 years, showing the association of NLR across different age groups.

A linear correlation analysis between age and NLR in the health examination population showed a correlation coefficient of r = 0.10, indicating no significant linear relationship between age and NLR (**Figure 2A**). Further stratification by age groups (<18, 18-40, 41-60, >61 years) revealed a trend of increasing mean NLR values with advancing age. The <18 group had the lowest mean NLR (1.59), while the >61 group had the highest mean NLR (1.99). Multiple comparison tests showed significant differences between the <18 group and the 18-40, 41-60, and >61 groups (*P* < 0.0001), with higher mean NLR values observed in older age groups. The maximum mean difference between groups was 0.39. Significant differences were also observed between the 18-40 group and the 41-60 and >61 groups (*P* < 0.0001), with higher mean values in the older groups. However, no significant difference was found between the 41-60 and >61 groups (*P* > 0.10) (**Figure 2B**)

### Preliminary confirmation of NLR as an independent risk factor and its predictive ability for post-trauma infections

LASSO univariate analysis identified 14 variables as potential predictors of post-trauma infections, including NLR, age, white blood cell count, hemoglobin level, platelet count, absolute lymphocyte count, hypertension, diabetes, cerebral infarction, coronary artery disease, smoking, alcohol consumption, urinary catheterization, and mechanical ventilation (P < 0.05) (**Supplementary Figure 2**).

**Figure 3:**
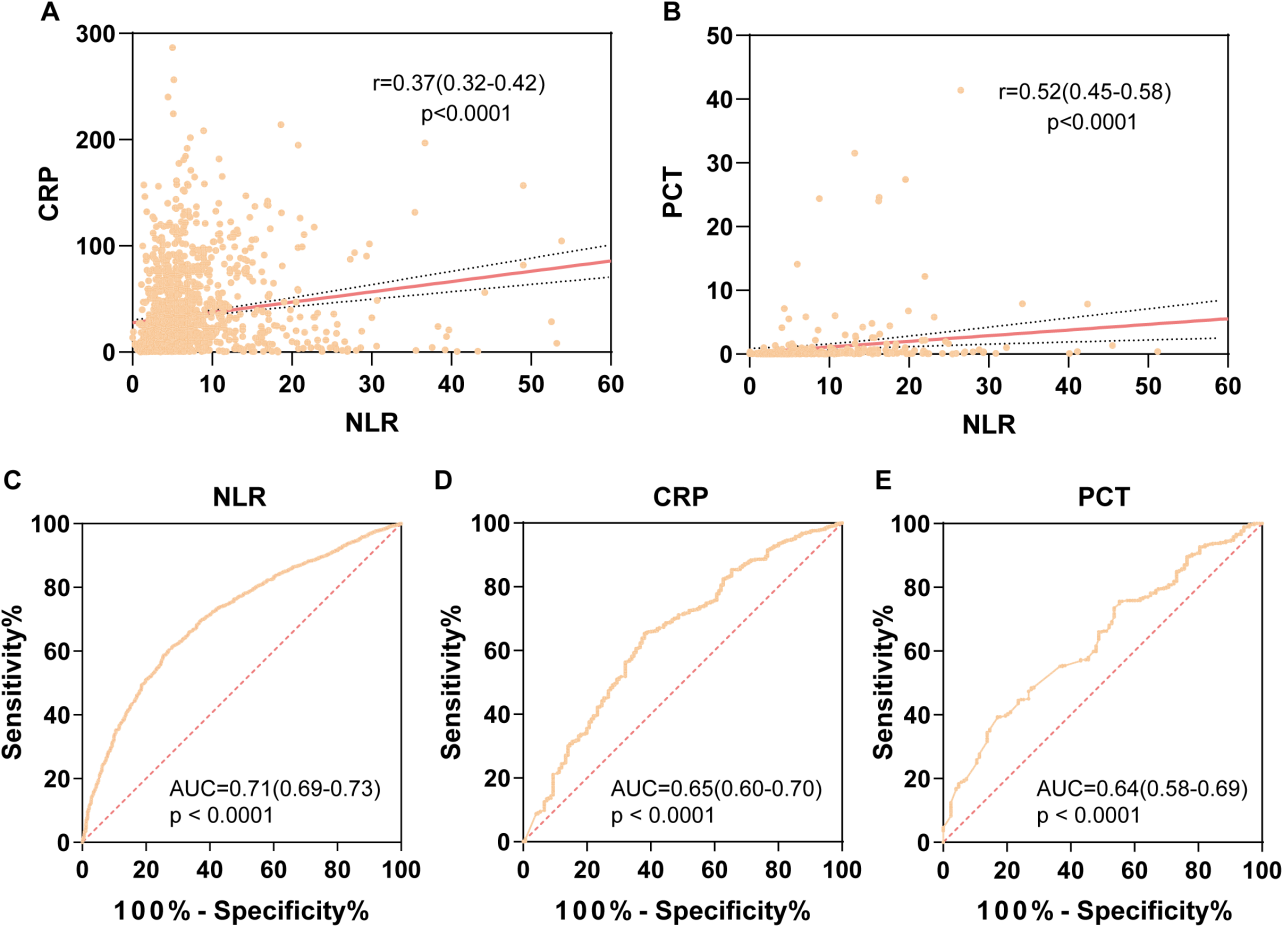
Correlation and predictive value of NLR compared with CRP and PCT. **(A)** Correlation analysis between NLR and CRP, with correlation coefficient r = 0.37, *p* < 0.0001. **(B)** Correlation analysis between NLR and PCT, with correlation coefficient r = 0.52, *p* < 0.0001 (r > 0.3 indicates weak correlation; r between 0.3 and 0.7 indicates moderate correlation strength). **(C)** ROC curve of NLR for predicting post-traumatic infection, with AUC = 0.71 (95% CI: 0.69-0.73). **(D)** ROC curve of CRP for predicting post-traumatic infection, with AUC = 0.65 (95% CI: 0.60-0.70). **(E)** ROC curve of PCT for predicting post-traumatic infection, with AUC = 0.64 (95% CI: 0.58-0.69).

Further multivariate logistic regression analysis confirmed 10 variables, including NLR, as independent risk factors for post-trauma infections (*P* < 0.05). The odds ratios (OR) and 95% confidence intervals (CI) for these variables were as follows: NLR, 1.43 [1.18-1.73]; age, 0.02 [0.02-0.04]; white blood cell count, 1.37 [1.14-1.64]; absolute lymphocyte count, 0.81 [0.68-0.97]; hypertension, 1.26 [1.07-1.49]; diabetes, 1.32 [1.09-1.59]; cerebral infarction, 1.66 [1.30-2.12]; coronary artery disease, 0.68 [0.47-0.97]; urinary catheterization, 2.75 [2.34-3.24]; and mechanical ventilation, 1.52 [1.14-2.01] (**Table 3**) NLR showed a weak positive correlation with CRP (r = 0.37 [0.32-0.42], **Figure 3A**) and a moderate positive correlation with PCT (r = 0.52 [0.45-0.58], **Figure 3B**). ROC curve analysis demonstrated that NLR had an area under the curve (AUC) of 0.71 (95% CI: 0.69-0.73, P < 0.0001) for predicting infections, with a cutoff value of 4, sensitivity of 60.28% (95% CI: 59.24-61.31), and specificity of 72.85% (95% CI: 69.71-75.77) (**Figure 3C**). In comparison, the AUC for CRP was 0.65 (95% CI: 0.60-0.70), with a cutoff value of 29.68, sensitivity of 65.34% (95% CI: 62.00-67.00), and specificity of 62.00% (95% CI: 54.00-69.00) (**Figure 3D**). For PCT, the AUC was 0.64 (95% CI: 0.58-0.69), with a cutoff value of 0.07, sensitivity of 39.38% (95% CI: 35.00-44.00), and specificity of 82.93% (95% CI: 75.00-89.00) (**Figure 3E**).

**Table 3:** Multivariate logistic analysis of factors associated with post-traumatic nosocomial infection.

Variable	Multivariate logistic analysis					
	β	Odds ratio (95% CI)	Std. Error	z value	*P* value	
Intercept	-3.69	0.02 [0.02-0.04]	0.21	-17.63	< 0.001	***
Age	1.38	3.99 [3.34-4.77]	0.09	15.16	< 0.001	***
WBC	0.31	1.37 [1.14-1.64]	0.09	3.36	< 0.001	***
HB	-0.03	0.97 [0.83-1.14]	0.08	-0.39	0.694	
PLT	-0.19	0.83 [0.59-1.17]	0.17	-1.11	0.267	
LY	-0.21	0.81 [0.68-0.97]	0.09	-2.36	0.019	*
NLR	0.36	1.43 [1.18-1.73]	0.10	3.71	< 0.001	***
Hypertension	0.23	1.26 [1.07-1.49]	0.08	2.75	0.006	**
Diabetes	0.28	1.32 [1.09-1.59]	0.10	2.90	0.003	***
Cerebral_infarction	0.51	1.66 [1.30-2.12]	0.12	4.10	< 0.001	***
CHD	-0.39	0.68 [0.47-0.97]	0.19	-2.10	0.036	*
Smoking	0.13	1.14 [0.83-1.55]	0.16	0.82	0.412	
Drinking	0.25	1.29 [0.93-1.78]	0.17	1.53	0.127	
Catheterization	1.01	2.75 [2.34-3.24]	0.08	12.12	< 0.001	***
Ventilator	0.42	1.52 [1.14-2.01]	0.15	2.87	0.004	**

Signif.codes: 0.001‘* * *’, 0.01‘* *’, 0.05‘*’

### Subgroup analysis of NLR and post-trauma infections by age group

To further assess the value of NLR in post-trauma populations, we conducted subgroup analyses by age, dividing the participants into three groups: 18-40 years, 41-60 years, and >60 years. LASSO univariate and multivariate logistic regression analyses were performed within each group. 18-40 years: Independent risk factors for post-trauma infections included diabetes (OR 8.39 [2.04-27.94], *P* = 0.001), smoking (OR 2.82 [1.36-5.53], *P* = 0.004), and mechanical ventilation (OR 3.84 [1.55-9.31], *P* = 0.003). NLR (*P* = 0.195) was not identified as an independent risk factor in this age group. 41-60 years: Independent risk factors included red blood cell count (OR 0.57 [0.39-0.85], *P* = 0.005), diabetes (OR 2.31 [1.40-3.67], *P* = 0.001), cerebral infarction (OR 4.67 [1.93-10.30], *P* < 0.001), urinary catheterization (OR 1.81 [1.20-2.75], *P* = 0.005), and mechanical ventilation (OR 1.84 [1.07-3.09], *P* = 0.024). NLR (*P* = 0.394) was also not identified as an independent risk factor in this group. >60 years: Independent risk factors included NLR (OR 1.43 [1.16-1.78], *P* = 0.001), white blood cell count (OR 1.53 [1.24-1.88], *P* < 0.001), absolute lymphocyte count (OR 0.78 [0.64-0.95], *P* = 0.014), hypertension (OR 1.24 [1.04-1.47], *P* = 0.017), cerebral infarction (OR 1.60 [1.24-2.05], *P* < 0.001), and urinary catheterization (OR 3.04 [2.54-3.65], *P* < 0.001). In this group, NLR was confirmed as an independent risk factor for post-trauma infections (**Table 4**).

**Table 4:** Age-based subgroup multivariate logistic regression analysis.

Variable	18-40 years				41-60 years				> 61 years			
	β	OR	95% CI	*P*	β	OR	95% CI	*P*	β	OR	95% CI	*P*
Intercept	-3.91	0.02	0.00-0.05	< 0.001	-2.92	0.05	0.03-0.10	< 0.001	-2.62	0.07	0.06-0.09	< 0.001
Sex									0.1	1.11	0.93-1.32	0.26
WBC	0.2	1.23	0.59-2.51	0.58					0.42	1.53	1.24-1.88	< 0.001
RBC	-0.56	0.57	0.20-1.76	0.315	-0.56	0.57	0.39-0.85	0.005				
HB	-0.44	0.64	0.22-1.92	0.436								
PLT												
LY					-0.37	0.69	0.44-1.08	0.105	-0.25	0.78	0.64-0.95	0.014
MO												
NE												
NLR	0.49	1.64	0.77-3.45	0.195	0.19	1.21	0.77-1.89	0.394	0.36	1.43	1.16-1.78	0.001
Hypertension	0.59	1.81	0.36-6.64	0.422					0.21	1.24	1.04-1.47	0.017
Diabetes	2.13	8.39	2.04-27.94	0.001	0.84	2.31	1.40-3.67	0.001	0.14	1.15	0.94-1.41	0.177
CI	2.3	9.94	0.36-271.54	0.119	1.54	4.67	1.93-10.30	< 0.001	0.47	1.6	1.24-2.05	< 0.001
CHD									-0.31	0.73	0.50-1.05	0.1
Smoking	1.04	2.82	1.36-5.53	0.004								
Drinking												
Catheterization	0.42	1.52	0.72-3.10	0.261	0.59	1.81	1.20-2.75	0.005	1.11	3.04	2.54-3.65	< 0.001
Ventilator	1.35	3.84	1.55-9.31	0.003	0.61	1.84	1.07-3.09	0.024				

### Subgroup analysis of NLR and post-trauma infections by gender

First, we focused on the female population, with the occurrence of post-trauma infection as the outcome variable. Potential influencing factors were initially screened using LASSO regression and univariate analysis, including: “Age,” “WBC,” “HB,” “PLT,” “LY,” “MO,” “NLR,” “Hypertension,” “Diabetes,” “Cerebral Infarction,” “Coronary Heart Disease,” “Smoking,” and “Catheterization.” These factors were subsequently included in a multivariate logistic regression analysis. The results indicated that NLR (*P* < 0.001) was an independent influencing factor for post-trauma infections, with a regression coefficient of 0.41 and an OR with 95% CI of 1.50 (1.18, 1.91). In the female population, the area under the ROC curve (AUC) for NLR alone in predicting post-trauma infections was 0.63 (95% CI: 0.60, 0.65).

Similarly, we analyzed the male population. After univariate analysis, potential influencing factors included: “Age,” “WBC,” “HB,” “LY,” “MO,” “NLR,” “Hypertension,” “Diabetes,” “Cerebral Infarction,” “Coronary Heart Disease,” “Smoking,” “Alcohol Consumption,” “Catheterization,” and “Ventilator.” These factors were also subjected to multivariate logistic regression analysis. The results demonstrated that NLR (*P* < 0.001) was an independent influencing factor for post-trauma infections, with a regression coefficient of 0.49 and an OR with 95% CI of 1.63 (1.24, 2.12). The AUC for NLR alone in predicting the risk of post-trauma infections in the male population was 0.64 (95% CI: 0.61, 0.66).

### NLR characteristics in different infection sites and fungal coinfections

**Figure 4:**
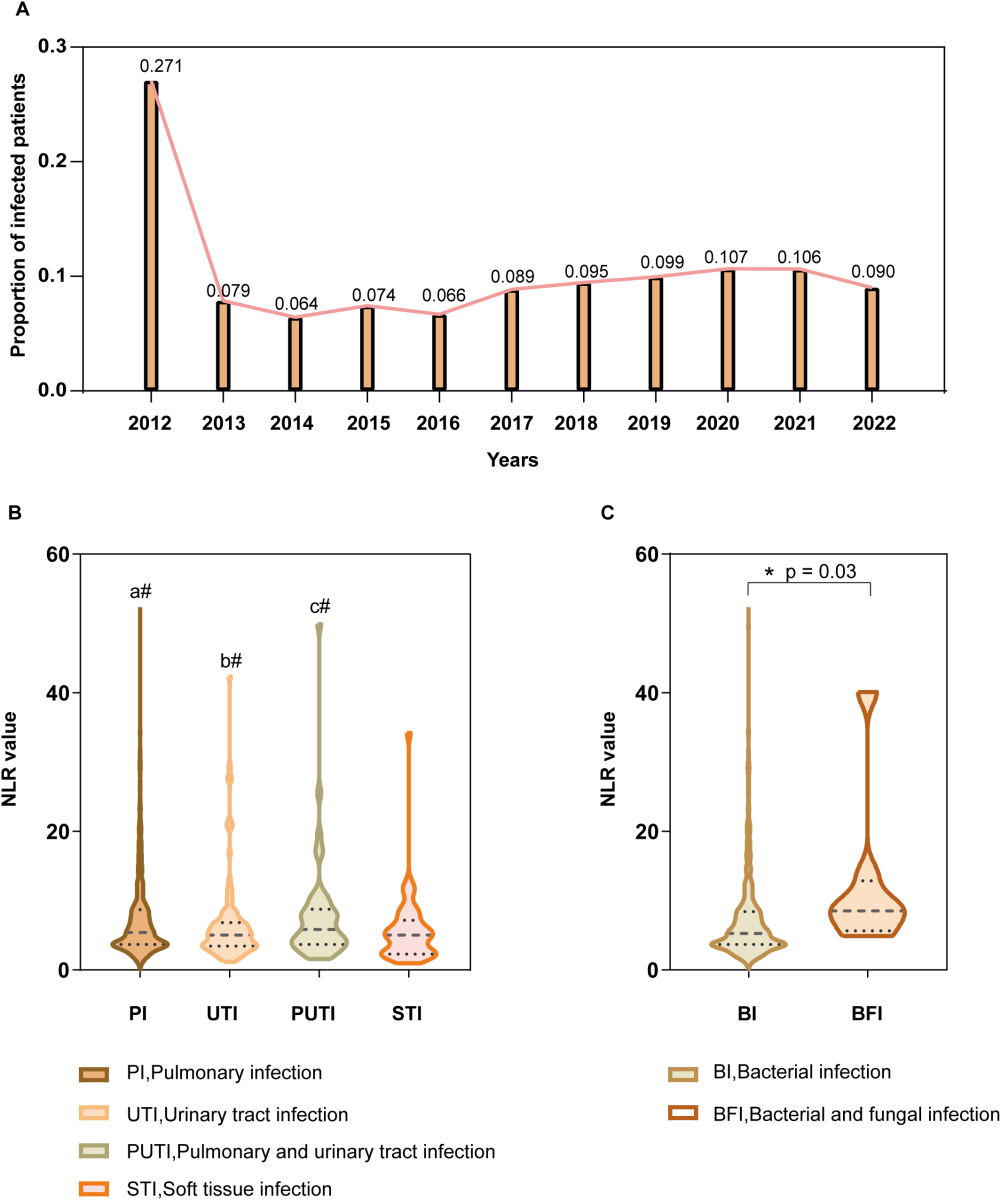
Subgroup Analysis **(A)** Annual percentage of hospital-acquired infections among hospitalized acute trauma patients over 11 consecutive years at this center (Y-axis), with corresponding years on the X-axis. **(B)** NLR values by infection site (Y-axis) for pulmonary, urinary tract, combined pulmonary and urinary, and soft tissue infections (X-axis). a#(*P*>0.05), PI vs UTI, PI vs PUTI, PI vs STI; b#(*P*>0.05), UTI vs PUTI, UTI vs STI; c#(*P*>0.05), PUTI vs STI. **(C)** NLR values (Y-axis) in patients with and without fungal co-infections (X-axis). Asterisk (*) denotes statistical significance at P ≤ 0.05.

Data from 11 consecutive years at the National Center for Trauma Medical were analyzed, showing that the annual infection rate among trauma patients ranged from 6.4% in 2014 to 27.1% in 2012, with an average infection rate of 12.8% (**Figure 4A**). Subgroup analyses were conducted based on infection sites, which were categorized into five groups: pulmonary infections, urinary tract infections, combined pulmonary and urinary tract infections, soft tissue infections, and other infections. NLR values by infection site: Pulmonary infections: median NLR 7.45 (IQR: 3.66-8.69), Urinary tract infections: median NLR 6.66 (IQR: 3.43-6.83), Combined pulmonary and urinary tract infections: median NLR 7.85 (IQR: 3.66-8.75), Soft tissue infections: median NLR 5.76 (IQR: 2.29-7.18). No statistically significant differences in NLR values were observed among these four groups (*P* > 0.05, **Figure 4B**). The “other infections” group was excluded from comparisons due to heterogeneity in infection causes and types. NLR and fungal coinfections: The median NLR for post-trauma infection patients without fungal involvement was 7.36 (IQR: 3.66-8.41), while for those with fungal coinfections, the median NLR was 12.34 (IQR: 5.66-12.87). Comparison of the two groups showed a statistically significant difference (*P* = 0.03), with NLR values significantly higher in patients with fungal coinfections (**Figure 4C**).

## Discussion

This study provides a single-center, large-scale analysis of the normal reference range for NLR, establishing reference values for healthy individuals and, for the first time, defining a separate NLR reference range for minors. The adult NLR reference range established in this study represents the population in southwestern China. Compared with two existing studies on populations from coastal and northern regions of China (Meng et al., 2018; Wu et al., 2019), our results show higher 95% reference ranges across both age and gender groups. This discrepancy may be partially attributed to differences in geographical and environmental factors, as well as potential immune alterations in the population following COVID-19 infection. Therefore, our NLR findings may more accurately reflect the current immune status of the population.

We further introduced the neutrophil-to-lymphocyte ratio (NLR) into the analysis of post-trauma infections, complementing existing early predictive indicators for post-trauma infection. The rationale for selecting NLR lies in its ability to capture systemic changes during systemic infection and sepsis, where all blood cells are activated, leading to significant alterations in cell counts, functionality, receptor expression, and secretion of signaling molecules and humoral substances. NLR effectively reflects these changes (Aird, 2003), and its sensitivity in diagnosing various infections and sepsis has been validated in multiple studies (Farkas, 2020; Marik and Stephenson, 2020; Ham et al., 2020). As a rapid and effective immunological biomarker, NLR not only reflects stress levels, injury, inflammation, and disease severity (Zahorec, 2017), but also demonstrates strong predictive value for the occurrence of sepsis and mortality outcomes in ICU patients (Akilli et al., 2014; Salciccioli et al., 2015; Hwang et al., 2017). Analysis of trauma-related infection data from our center over 11 years shows that the infection rate peaked at 27.1% in 2012, the highest during the study period, before dropping to 7.9% in 2013. From 2013 to 2020, a slight upward trend was observed, with the largest annual increase reaching 4.5%. However, from 2020 to 2022, the infection rate showed a gradual decline, with an average annual decrease of approximately 1.7%. Notably, during this period, the proportion of severely injured patients (ISS ≥ 16) increased. These findings suggest that efforts to control trauma-related infections at our center have been effective overall.

Despite these improvements, post-trauma infections remain a significant challenge, with infection rates still reaching approximately 9% in 2022. Patients with post-trauma infections had significantly longer hospital stays and higher medical costs compared to non-infected patients, with an average increase in hospital stay of 6.61 days and additional costs of approximately 33,883 CNY per patient. These infections not only reduce bed turnover rates but also impose substantial economic burdens on individuals and healthcare systems. Our findings align with existing research trends on nosocomial infections, and the mortality rate of trauma patients with infections was approximately eight times higher than that of the control group. Post-trauma infections remain a persistent and critical issue requiring solutions. The introduction of NLR as a predictive indicator provides a new approach and perspective for infection control, with the potential to enhance early detection and management strategies for post-trauma infections. This study establishes NLR as an independent risk factor for post-trauma infections through multivariate analysis. However, considering the age-related changes in the immune system, such as reduced Toll-like receptor function, chemotaxis, phagocytosis, cytokine production, and thymic involution leading to a decline in naive T cells and effector memory T cell subsets, the older adult population experiences a decline in natural killer cell cytotoxicity and cytokine function. These changes increase the risk and severity of infections in older adults (Tuano et al., 2021). Based on this, we hypothesized that the significance of NLR in post-trauma infections might differ across age groups. To test this, we conducted subgroup analyses by age. The results showed that NLR was not an independent risk factor across all age groups but was significantly associated with post-trauma infection risk in patients aged over 60, confirming our hypothesis. Additionally, we identified other independent risk factors for post-trauma infections, including white blood cell count at admission, age, hypertension, diabetes, cerebral infarction, coronary artery disease, urinary catheterization, and mechanical ventilation. These variables have been previously reported in the literature, and our study validates their associations. However, the focus of this study was on the value of NLR in post-trauma infections, and these factors were not analyzed in detail (Dvorak et al., 2024).

Following the confirmation of NLR as an independent risk factor, we further evaluated its predictive ability for infections in trauma patients. ROC curve analysis yielded an AUC of 0.71, indicating good predictive performance, with a cutoff value of 4. This cutoff exceeds the upper limit of the NLR reference range established in our study, highlighting its clinical significance. To further emphasize the utility of NLR in post-trauma infections, we compared it with commonly used inflammatory markers, such as CRP and PCT. NLR showed a positive but weak correlation with CRP and a stronger positive correlation with PCT. Additionally, the ROC AUCs for CRP and PCT in predicting post-trauma infections were 0.65 (95% CI: 0.60-0.70) and 0.64 (95% CI: 0.58-0.69), respectively, both lower than that of NLR. This suggests that NLR outperforms CRP and PCT in predicting post-trauma infections, consistent with findings in critically ill populations reported in previous studies (Gürol et al., 2015)

Despite the promising value of NLR in predicting post-trauma infections and assessing disease severity, our findings suggest that NLR cannot differentiate between primary infection sites or causative pathogens. Previous studies have confirmed that the predominant bacterial species vary by infection site, such as pulmonary, urinary, and soft tissue wound infections, and these differences significantly impact prognosis (Xie et al., 2020). However, in our study, NLR values showed no significant differences among patients with pulmonary infections, urinary tract infections, combined pulmonary and urinary infections, or soft tissue infections. This indicates that while NLR can predict post-trauma infections and serves as an independent risk factor, it may not differentiate between bacterial species. Further detailed research is needed to address this limitation.

In addition to exploring bacterial infections, we analyzed fungal coinfections. Existing studies report a fungal infection rate of 2.6% in patients with injuries from explosive blasts (Fares et al., 2013) and approximately 3% in other specific trauma studies. These studies, however, are limited to specific trauma types and may not reflect broader trauma populations (Fares et al., 2013; Moran and Shin, 2006a; Obradović-Tomasev et al., 2016). In our comprehensive analysis of trauma patients, we observed a fungal infection rate of approximately 0.8‰, significantly lower than previous studies. This discrepancy may be due to the broader scope of our study population compared to the narrow focus of previous research. When examining the incidence of specific fungal species, we found that populations with impaired immune function exhibited increased rates of fungal infections, whereas those with normal immune function did not. This suggests that fungal infections are associated with impaired immunity. Our findings also confirmed that patients with fungal coinfections had significantly higher NLR values than those without fungal infections, consistent with the general pattern of fungal infections (Skiada et al., 2011; Lelievre et al., 2014). This supports the utility of NLR as a marker for immune function status (Korkmaz et al., 2015).

### Limitations

This study has several limitations. First, while we established NLR reference intervals for healthy populations, categorizing them into adults and minors, the lack of data prevented us from stratifying or adjusting for factors such as pre-existing comorbidities, medication use, or lifestyle. Second, due to the limited number of viral infection cases, we did not analyze NLR values in bacterial versus viral infections. Third, the independent variables included in this study may not fully encompass all risk factors associated with post-trauma infections, as the primary focus was on the value of NLR, and a comprehensive analysis of other risk factors was not conducted. Fourth, the study population consisted of hospitalized patients with acute trauma, excluding many trauma-related cases, which may introduce certain biases. Additionally, as this is a single-center study, validation with data from other domestic and international trauma centers would enhance the persuasiveness of the findings. Furthermore, although we observed a significant increase in NLR values in patients with fungal infections compared to those with bacterial infections alone, the small sample size of fungal infection cases limits the stability of these results, necessitating further support from larger and more detailed datasets. Finally, given the unique characteristics of the trauma population included in this study-such as the limited clinical assessment of immunosuppression, the relatively low proportion of individuals with chronic inflammatory diseases, and the rare use of steroid treatments-we did not further analyze the potential confounding effects of these factors.

## Conclusion

The 2.5th-97.5th percentile range for NLR in this study was established as 0.69-3.48 for minors and 0.86-3.83 for adults. The reference range expands with increasing age, and females exhibit slightly higher reference ranges compared to males. NLR was identified as an independent risk factor for post-trauma infections, with its predictive value being particularly significant in patients over 60 years of age. Although NLR cannot differentiate between infection sites or pathogen types, elevated NLR values may serve as a useful indicator for the occurrence of fungal infections

## Data Availability

The original contributions presented in the study are included in the article/**Supplementary Material**. Further inquiries can be directed to the corresponding author/s.
