# Supplementary figures and images for: Reference range of neutrophil-to-lymphocyte ratio in healthy individuals and its predictive value for post-trauma nosocomial infections
Source: Front Cell Infect Microbiol. 2025 Apr 22;15:1529532. doi: 10.3389/fcimb.2025.1529532 (PMC12053171; doi:10.3389/fcimb.2025.1529532)

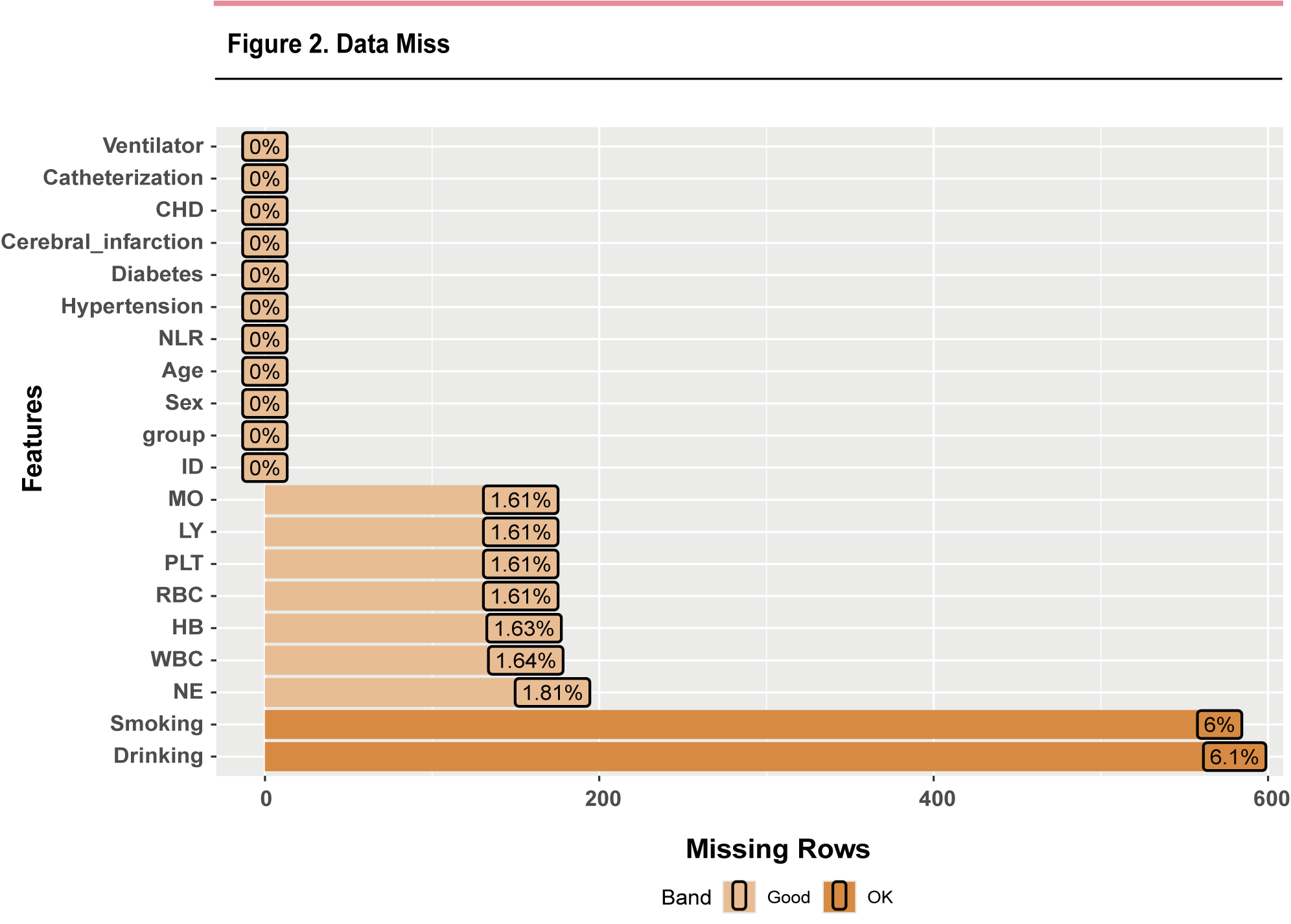

Supplement: Supplementary file 2 [file Image1.tif]

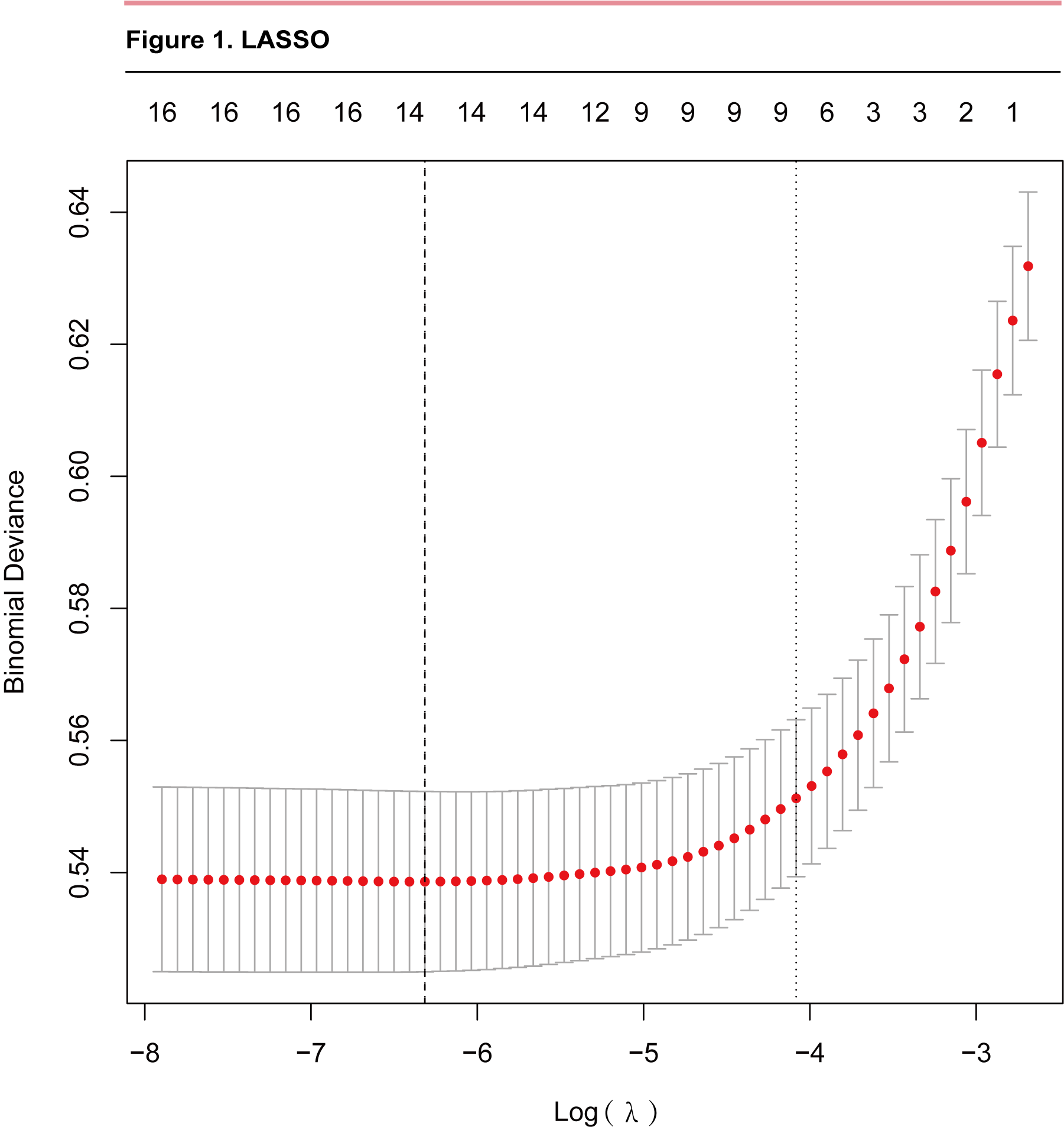

Supplement: Supplementary file 3 [file Image2.tif]
